# Drug Target Investigation of *N*-*p*-Coumaroyl-*N*’-Caffeoylputrescine, a Naturally-Occurring Alkaloid Derived from *Saxifraga tangutica*

**DOI:** 10.3390/antiox14010012

**Published:** 2024-12-25

**Authors:** Chuang Liu, Jun Dang, Minchen Wu

**Affiliations:** 1 School of Biotechnology, Jiangnan University, Wuxi 214122, China; 7210201054@stu.jiangnan.edu.cn; 2 Key Laboratory of Tibetan Medicine Research, Northwest Institute of Plateau Biology, Chinese Academy of Sciences, Xining 810000, China

**Keywords:** *Saxifraga tangutica*, *N*-*p*-coumaroyl-*N*’-caffeoylputrescine, drug affinity responsive target stability, reverse virtual docking, molecular dynamics simulation

## Abstract

The exploration of drug targets has always been a priority in new drug research, and this work is even more essential for natural active compounds. *Saxifraga tangutica* is a traditional Tibetan medicine with excellent antioxidant properties. In this study, an alkaloid, *N*-*p*-coumaroyl-*N*’-caffeoylputrescine (PCC), was first isolated from the plant, *Saxifraga tangutica*, with a DPPH scavenging rate of 0.936 μg/mL. To further identify its target, the drug affinity responsive target stability technique and multiple public databases were integrated to retrieve a total of 317 common targets from comprehensive screening. A further bioinformatics analysis not only identified 13 hub targets but also indicated PCC as having biological activities against cancer and affecting metabolic diseases. Integrating reverse virtual docking, molecular dynamics simulations, and cellular thermal shift assays ultimately focused on HSP90AA1 as the target of PCC. An in vitro study on liver (HepG2) cells and breast (MCF-7) cancer cells revealed that PCC modulates HSP90AA1, subsequently affecting Mut-p53 expression, triggering a cascade effect that reduced adriamycin-induced drug resistance in cells. Furthermore, a prediction of the absorption, distribution, metabolism, excretion, and toxicity was also applied to evaluate the drug-like properties of PCC. Overall, the integrated strategy used in this study successfully identified the target of PCC, providing a valuable paradigm for future research on the action targets of natural products.

## 1. Introduction

Novel small-molecule compounds found in nature are a vital source of inspiration for new drug development, and the continual advancement in biochemical techniques has significantly improved the ability to identify the drug targets of these compounds [1,2]. Drug affinity responsive target stability (DARTS) is designed to identify protein targets of small-molecule ligands without altering their structure [3]. During this process, the binding affinity between the ligand and its target is a pivotal factor. The formation of ligand-protein complexes alter the original proteolytic susceptibility, and the involvement of nonspecific proteases (e.g., subtilisin, thermolysin, pronase) will maximize the screening of these complexes. Finally, high-sensitivity mass spectrometry enables label-free target identification. Considering the inherent advantages of the low cost and high efficiency of DARTS, it has been widely applied in the study of unknown mechanisms and target identification of bioactive natural products [4,5].

In contrast to the traditional “one molecule-one target-one disease” paradigm, scientists now generally recognize that “drug-target-disease” interactions are a complex regulatory network [6,7]. On the basis of the above concept, several online databases have been established for drug target prediction, and their reliability has been recognized by scientists [8]. These online platforms are primarily based on pharmacophore models and molecular fingerprinting techniques, leveraging large datasets for training and learning to generate a high-confidence potential target database for compounds with unknow activity [9,10,11,12]. In addition, the construction of protein-protein interaction (PPI) networks and the analysis of bioinformatics further explains the key targets through which small molecule drugs exert their biological functions, as well as the complex relationships (e.g., pharmaceutical efficacy, side effects, and off-target effects) among them [13].

Currently, combining traditional wet experimental methods with dry experimental means that rely on computers is a more widespread sight in drug research. Owing to the rapid advancements of computer performance, virtual docking has gained widespread acceptance for preliminary drug target discovery, which is more efficient and cost-controlled [14,15]. Under the concept of “one molecule-multiple targets”, reverse virtual screening allows the simultaneous docking of multiple proteins with a small molecule to rapidly identify complexes with a high binding affinity to it [16,17]. Through a molecular dynamics simulation, we can further elucidate the binding mode of small molecules and protein targets, as well as the dynamic changes within the complex [18], thereby improving the hit rate of subsequent wet experiments.

Reactive oxygen (ROS) and reactive nitrogen (RNS) are natural byproducts of cellular metabolism. When their production and consumption become imbalanced, oxidative stress occurs. Long-term oxidative stress has been shown to contribute to the onset and progression of numerous diseases [19,20]. HSP90AA1, a highly conserved molecular chaperone protein with hundreds of client proteins, undergoes misfolding, unfolding, and aggregation under stress conditions (e.g., temperature, oxidative stress), disrupting the function of its client proteins. Given the physiological roles of these client proteins, HSP90AA1 is closely linked to various diseases (e.g., cancer, neurodegenerative diseases) [21,22]. Thus, the exploration of HSP90AA1 inhibitors often leads to unexpected comprehensive therapeutic effects.

In this study, *N*-*p*-coumaroyl-*N*’-caffeoylputrescine (PCC), an alkaloid antioxidant, was first isolated from *Saxifraga tangutica* (*S. tangutica*), and a comprehensive strategy of wet and dry experiments was employed to further investigate its bioactivity and protein targets. This approach not only reveals the potential high-value applications of PCC, but also offers a methodological reference for the application of natural products in drug development.

## 2. Materials and Methods

### 2.1. Preparation and Characterization of PCC

The whole plants of *S. tangutica* were found in the Guoluo Tibetan Autonomous Prefecture (99°47′35″ E, 34°33′37″ N), Qinghai, China. A voucher specimen (No. 0325734) was deposited at the Qinghai-Tibet Plateau Museum of Biology. Five hundred grams of dried whole plants was extracted three times with 12 L of methanol (Kelon Chemical Reagent Factory, Chengdu, Sichuan, China), concentrated to 500 mL by a rotary evaporator, mixed with 200 g of polyamide, and then transferred to an oven for drying, to obtain 265 g of sample-polyamide mixture. The mixture was pretreated by connecting an MCI column (Mitsubishi, Tokyo, Japan) to an MPLC (Hanbon Science & Technology Co., Ltd., Huaian, Jiangsu, China) system, with the methanol concentration gradually increased from 0 to 100% over 120 min, followed by 30 min of elution with 100% methanol. Six fractions were eventually recovered, and the target fraction 4 (Fr4) was mixed with 20.4 g of polyamide and dried, pending the next procedure.

A Spherical C18 column (SiliCycle, Quebec, QC, Canada) was connected to an MPLC system for the preparation of Fr4. The elution program was as follows: 0–90 min with 18–26% acetonitrile, followed by 20 min with 26% acetonitrile. Five fractions were finally collected, with the target fractions 5 (Fr4-5) dissolved in 12 mL of methanol, awaiting further processing. A Click XIon column (Acchrom Corporation, Beijing, China) was connected to an HPLC system for the preparation of Fr4-5. The isocratic elution with 91% acetonitrile for 30 min yielded the target fraction Fr4-5-1. The fraction was concentrated under reduced pressure and weighed, yielding 678.6 mg.

The ESI-MS data of Fr4-5-1 was recorded with a Waters QDa electrospray ionization mass spectrometer (Waters Instruments Co., Milford, MA, USA), and the NMR data were collected with a Bruker Avance 600 MHz instrument (Bruker, Billerica, MA, USA).

### 2.2. Assay of the Antioxidant Capacity

The antioxidant activity of PCC has been evaluated via the DPPH method, which is based on the literature, with minor modifications [23]. A 0.3 mg/mL DPPH (Merck, Darmstadt, Hesse, Germany) solution was prepared in ethanol for use. Different concentrations (0, 0.1, 1, 5, 10, 25, 50, and 100 μg/mL) of PCC solution (30 μL) were mixed with DPPH solution (70 μL) and incubated for 30 min in the dark. Then, the absorbance of these solutions was read at 517 nm via a microplate reader. To ensure the stability, this experiment was repeated three times. The antioxidant capacity of PCC was calculated via the following equation:

DPPH scavenging activity (%) = [1 − (A_a_ − A_b_)/A_c_] × 100%

where A_a_ is the absorbance of the sample solution, A_b_ is the absorbance of a blank and Ac is the absorbance of the control, respectively.

### 2.3. Fishing of Protein Targets

#### 2.3.1. DARTS Assay

The cell lysates were incubated with or without PCC for 40 min at room temperature, followed by its treatment with pronase (Sigma-Aldrich, St. Louis, MO, USA) for 10 min. Subsequently, the protein information was analyzed via label-free proteomics and bioinformatics. *p* < 0.05 and a fold change >1.5 were used as the screening thresholds for potential protein targets.

#### 2.3.2. Online Database Probing

The 3D structure and SMILES string of PCC were uploaded to the following databases for target probing: TargetNet (http://targetnet.scbdd.com/, accessed on 3 May 2024) [11], Swiss Target Prediction (STP, http://www.swisstargetprediction.ch/, accessed on 3 May 2024) [24], PharmMapper (https://lilab-ecust.cn/pharmmapper/, accessed on 3 May 2024) [10], and Super-PRED (https://prediction.charite.de/, accessed on 3 May 2024) [12]. All the targets retrieved from the four databases were consolidated, and duplicate entries were removed to identify the potential targets of PCC. These targets were then standardized and annotated via the online UniProt database (https://www.uniprot.org/, accessed on 10 May 2024), with *Homo sapiens* selected as the species.

#### 2.3.3. Construction of the PCC Potential Targets Database

The results of the DARTS assay and online database probing were integrated, and a local database of potential PCC targets was constructed after removing duplicates. The STRING database (https://cn.string-db.org/, accessed on 10 May 2024) was used to analyze the protein-protein interaction (PPI) networks of these targets. The high-confidence interaction scores (0.7) were screened and output as tsv files, which were then visualized via Cytoscape 3.10.2. The hub targets were identified on the basis of the “Degree” values in the Cytohubba plugin, implying that the biological function of PCC is closely related to these targets.

### 2.4. Bioinformatics Analysis

Gene Ontology (GO) and Kyoto Encyclopedia of Genes and Genomes (KEGG) analyses, which are performed by the DAVID database (https://david.ncifcrf.gov/, accessed on 10 June 2024), are essential for exploring the molecular mechanisms of potential PCC targets [25]. Moreover, the hub targets were uploaded to the Diseases database (https://diseases.jensenlab.org/, accessed on 10 June 2024) [26], and the target-disease association profiles were derived from the text mining module of this database, with high-confidence results selected for visualization.

### 2.5. Reverse Virtual Docking

The pdb files of these hub targets with 3D coordinates were downloaded from the RCSB Protein Data Bank database (https://www.rcsb.org/, accessed on 3 August 2024), and some operations (e.g., removal of original ligands, charge assignment, and hydrogen addition) were implemented through PyMOL 3.1 and AutoDock 4. ChemBio3D 16.0 was used to map the 3D structure of the PCC and perform the energy minimization procedure. Then, AutoDock was used to further examine the hydrogen atoms, as well as the torsion bonds of PCC. After the AutoGrid program generated docking boxes, at least 100 docking runs were conducted with AutoDock to identify the optimal conformations. Finally, the results were visualized via ChimeraX 1.8 and LigPlot 2.2.

### 2.6. Molecular Dynamics Simulation

The open-source software GROningen MAchine for Chemical Simulations (GROMACS) 2023.2 was used to perform molecular dynamics simulations on the ligand-protein complex generated from reverse virtual docking. The protein force field was defined as CHARMM36, and the GAFF force field for the PCC was added via Sobtop 1.0 software [27]. A simulation box filled with TIP3P water molecules was constructed to encapsulate the complex. The energy minimization of the system was carried out using the steepest descent method. After minimization, isothermal-isochoric (NVT) and isothermal-isobaric (NPT) ensembles were applied to stabilize the temperature at 300 K and pressure at 1 bar. A 100 ns molecular dynamics simulation was then performed with a time step of 2 femtoseconds (fs), and simulation trajectories were saved every 10 picoseconds (ps) for further analysis. Root mean square deviation (RMSD) is used to measure the degree of deviation between the atomic coordinates and the reference coordinates. Hydrogen bond (H-bond) refers to the number of hydrogen bonds established by the simulated system during the simulation time. The Radius of gyration (Rg) is used to examine the average distance of individual atoms in the simulation system, relative to the center of mass of the molecule, which is an important parameter for measuring the size and compactness of the molecule. Root mean square fluctuation (RMSF) is used to quantify the degree of fluctuation in the positions of the atoms.

### 2.7. Cellular Thermal Shift Assay (CETSA)

An M-PER lysis buffer (Thermo Fisher, Waltham, MA, USA) was used to extract the total protein from the cells. The protein mixture was incubated with or without PCC for 30 min at room temperature. Next, the samples were evenly divided into 6 aliquots and heat-treated in a water bath at 50, 56, 62, 68, 74, and 80 °C for 3 min. After high-speed centrifugation (12,000 rpm, 3 min), the supernatant was collected for Western blot analysis to evaluate the HSP90AA1 levels.

### 2.8. In Vitro Experiments

#### 2.8.1. Cell Culture

For the experiment, 293T cells, HepG2 cells, and MCF-7 cells were purchased from Procell Life Science & Technology Co., Ltd. (Wuhan, China). Prior to further processing, the cells were cultured in DMEM, supplemented with 10% fetal bovine serum (Procell, Wuhan, China) and 1% penicillin-streptomycin (Servicebio, Wuhan, China). The conditions of the cell culture incubator were maintained at 37 °C with 5% CO_2_. Furthermore, the cell viability was measured according to the instructions of the CCK-8 kit (Vazyme, Nanjing, China).

#### 2.8.2. Western Blot Analysis

HepG2 and MCF-7 cells were inoculated at a density of 25 × 10^4^ cells per well in 6-well plates. After allowing the cells to adhere, they were incubated with varying concentrations of PCC (5, 10, and 30 μM) for 24 h. The total proteins from the different experimental groups were extracted via M-PER lysis buffer, and the protein content of each group was measured via a BCA kit (Vazyme, China).

Equal amounts of protein mixture were separated via 8% SDS-PAGE and transferred onto a PVDF membrane. The membrane was blocked with 5% skim milk for 2 h. After blocking, the PVDF membrane was further incubated with a primary antibody (14 h, 4 °C) and a secondary antibody (2 h, room temperature). Antibodies against HSP90AA1 and β-actin were purchased from Proteintech, Wuhan, China. Upon the completion of all the procedures, the target bands were visualized via an enhanced chemiluminescence (ECL) detection kit (Abbkine, Wuhan, China) and automated chemiluminescence detection equipment (Tanon, Shanghai, China). Furthermore, the semiquantitative analysis was realized via ImageJ software (imagej.net/ij/).

#### 2.8.3. qPCR Assay

HepG2 and MCF-7 cells were inoculated at a density of 25 × 10^4^ cells per well in 6-well plates. After the cells had adhered, they were incubated with adriamycin (ADR, 50 μM) and PCC (10 μM) for 16 h. Total RNA from each experimental group was extracted via RNA-easy isolation reagent (Vazyme, China). After the total RNA was converted to cDNA, it was further processed with 2 × AceQ qPCR SYBR Green Master Mix (Vazyme, China) for subsequent qPCR detection. The *Ct* value of β-actin was used as a benchmark, and the relative expression level of each group of mRNAs was represented by the value of 2^−ΔΔCt^. The detailed information on the primers used in this study is listed in Table 1.

### 2.9. Pharmacokinetic Profiling

The sdf structure file of PCC was uploaded to the ADMETlab 3.0 online platform (https://admetlab3.scbdd.com/, accessed on 23 August 2024) for predicting the pharmacokinetic properties. The ADMET characteristics primarily include absorption—Caco-2 permeability and human intestinal absorption (HIA); distribution—steady state volume of distribution (VDss) and blood-brain barrier (BBB); metabolism—human liver microsomal (HLM) stability, CYP1A2 inhibitor, CYP1A2 substrate, CYP2D6 inhibitor, CYP2D6 substrate, CYP3A4 inhibitor, and CYP3A4 substrate; excretion—CLplasma; toxicity—drug induced liver injury (DILI), rat oral acute toxicity, carcinogenicity, human hepatotoxicity, hematotoxicity, immunotoxicity, drug-induced nephrotoxicity, and drug-induced neurotoxicity.

### 2.10. Statistical Analysis

To ensure the reliability of the results in the in vitro experiments, all data were repeated three times. The final results were statistically analyzed and exported via a one-way analysis of variance (ANOVA) or *t*-tests from GraphPad Prism software (version 10, San Diego, CA, USA). Generally, a *p*-value < 0.05 indicated a statistically significant difference.

## 3. Results

### 3.1. Isolation and Characterization of the Compounds

**Figure 1:**
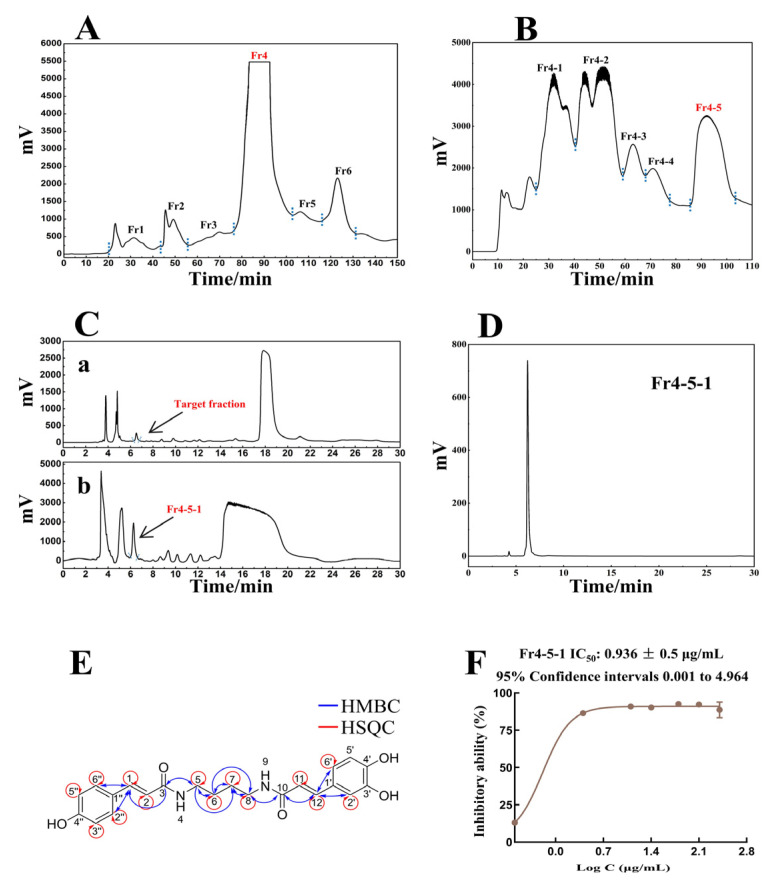
Pretreatment chromatogram of *S. tangutica* on MCI MPLC (**A**); preparation chromatogram of Fr4 on Spherical C18 MPLC (**B**); comparative chromatograms of the analysis (**Ca**) and preparation (**Cb**) of Fr4-5 on a Click XIon column; analytical chromatograms of Fr4-5-1 on a Click XIon column (**D**); chemical structure of PCC and two-dimensional correlation (**E**); and DPPH scavenging assay of PCC (**F**).

After pretreatment with MCI MPLC, the methanol extract of *S. tangutica* yielded six fractions (Figure 1A), with the target fraction Fr4 weighing 17.5 g. Subsequently, the Spherical C18 MPLC preparation separated five fractions from Fr4 (Figure 1B), with the target fraction Fr4-5 weighing 5.22 g. The 5.22 g sample was dissolved in methanol and subjected to further separation via a ClickXIon column (Figure 1C). After 24 cycles of preparation, Fr4-5-1 (PCC) was finally obtained, and 678.6 mg was weighed following concentration under reduced pressure. The purity verification diagram of PCC is shown in Figure 1D.

The dried PCC appeared as a yellowish solid, and the ESI-MS data for [M + Na]^+^ indicated a molecular mass of 396.17. In the ^1^H-NMR spectrum, the signal at *δ_H_* 7.97 corresponds to two protons, suggesting the presence of nitrogen atoms in PCC, whereas other hydrogen proton signals in the low field region (*δ_H_* 7.38, 6.93, 6.83, 6.79, and 6.74) indicate the presence of two aromatic rings. Additionally, signals at *δ_H_* 3.17 and 1.46 revealed the presence of alkyl fragments. In the ^13^C-NMR spectra, a total of 22 carbons were stacked, with a peak at *δ_C_* 165.3 indicating two carbonyl carbon atoms. In the HMBC spectrum, the correlation between *δ_C_* 129.1 and *δ_H_* 7.32 confirmed the relative position of a para-substituted aromatic ring, whereas the correlations between *δ_C_* 120.3, 113.8, and *δ_H_* 7.23 pinpointed the position of the hydrogen atom at carbon 12. In the HSQC spectrum, the correlations between *δ_C_* 38.3 and *δ_H_* 3.17, as well as between *δ_C_* 26.8 and *δ_H_* 1.46, allowed for the assignment of protons to their respective carbon atoms in the alkyl fragments. Finally, Fr4-5-1 was identified as *N*-*p*-coumaroyl-*N*’-caffeoylputrescine [28]. The detailed NMR signal assignments for PCC are listed in Table 2, with the structure shown in Figure 1E, and the corresponding spectral data provided in the Supporting Information (Figures S1–S5).

### 3.2. DPPH Scavenging Assay of PCC

The DPPH assay was conducted to assess the antioxidant activity of the highly purified PCC, then an inhibition activity curve was generated. As shown in Figure 1F, the concentration at which PCC achieved 50% inhibition (IC_50_) was determined to be 0.936 μg/mL, suggesting an excellent antioxidant capacity. On the basis of investigation, this is currently the most potent natural small molecule with antioxidant activity isolated by our team.

### 3.3. Screening Potential Targets of PCC

**Figure 2:**
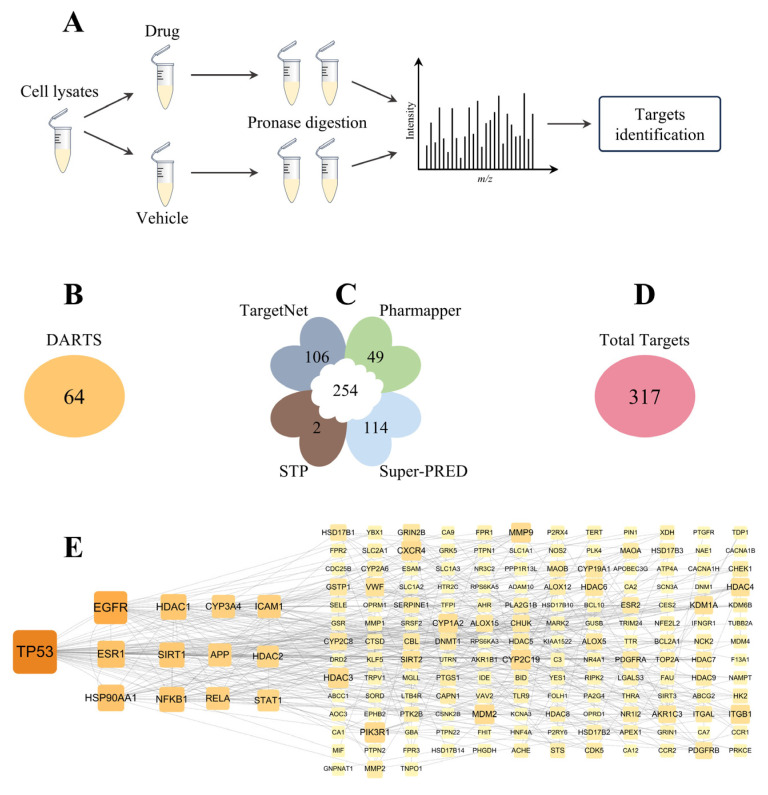
Protein target probing of PCC: flowchart of DARTS (**A**); number of targets identified by DARTS (**B**); number of targets retrieved from 4 open-source databases (**C**); number of potential targets for PCC (**D**); and identification of hub targets via Cytoscape analysis (**E**).

Label-free proteomics, based on DARTS, was used to screen for potential protein targets that interact with PCC (Figure 2A), resulting in 64 identified targets (Figure 2B). Additionally, 254 potential protein targets that interact with PCC were retrieved from four online network databases (Figure 2C). After normalization and the removal of duplicates, a total of 317 potential PCC targets were identified (Figure 2D), all of which were assumed to have possible interactions with PCC. The detailed information on these targets is presented in the Supporting Information (Table S1).

### 3.4. Hub Targets of PCC

Using the STRING database and Cytoscape software, 13 hub targets were identified from the 317 common targets (Figure 2E), including TP53, EGFR, ESR1, HSP90AA1, NFKB1, SIRT1, HDAC1, CYP3A4, APP, RELA, ICAM1, HDAC1, and STAT1.

### 3.5. Enrichment Analysis

**Figure 3:**
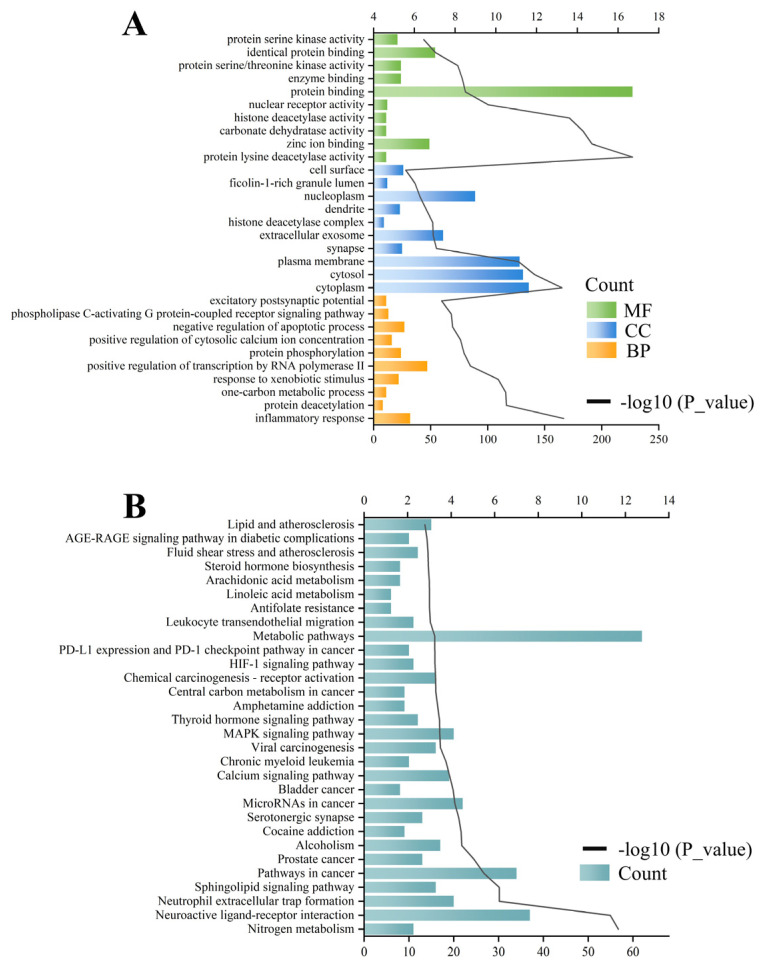
Result of the GO enrichment analysis of common targets (**A**); and KEGG enrichment analysis results of common targets (**B**).

The enrichment analysis of the 317 common targets was performed via the DAVID database. The GO results revealed that the common targets were associated with 157 molecular function (MF), 78 cellular component (CC), and 404 biological process (BP) terms. The top ten results for each of the GO terms were visualized and are presented in Figure 3A. BP was primarily associated with the inflammatory response, protein phosphorylation, and apoptosis. The CCs were mainly related to the cytoplasm, plasma membrane, and nucleoplasm. The MFs were associated with protein binding and kinase activity.

**Figure 4:**
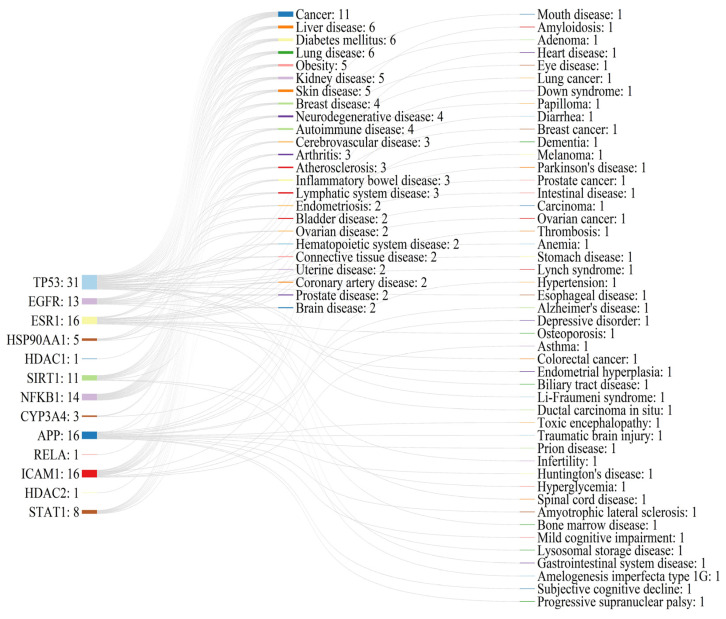
Text mining for disease research on hub targets.

The KEGG pathway enrichment analysis identified a total of 104 signaling pathways, and the top 30 pathways, after *p*-value ranking, were visualized and output (Figure 3B). As a result, many genes were enriched in metabolic pathways (hsa01100) and cancer pathways (hsa05200). Furthermore, on the basis of text mining of biomedical literature, an in-depth retrieval of the diseases associated with the 13 hub targets was conducted (Figure 4), which also supports the further investigation of the positive role that PCC plays in cancer (liver cancer and lung cancer) and metabolic diseases (diabetes mellitus and obesity).

### 3.6. Reverse Virtual Docking

**Figure 5:**
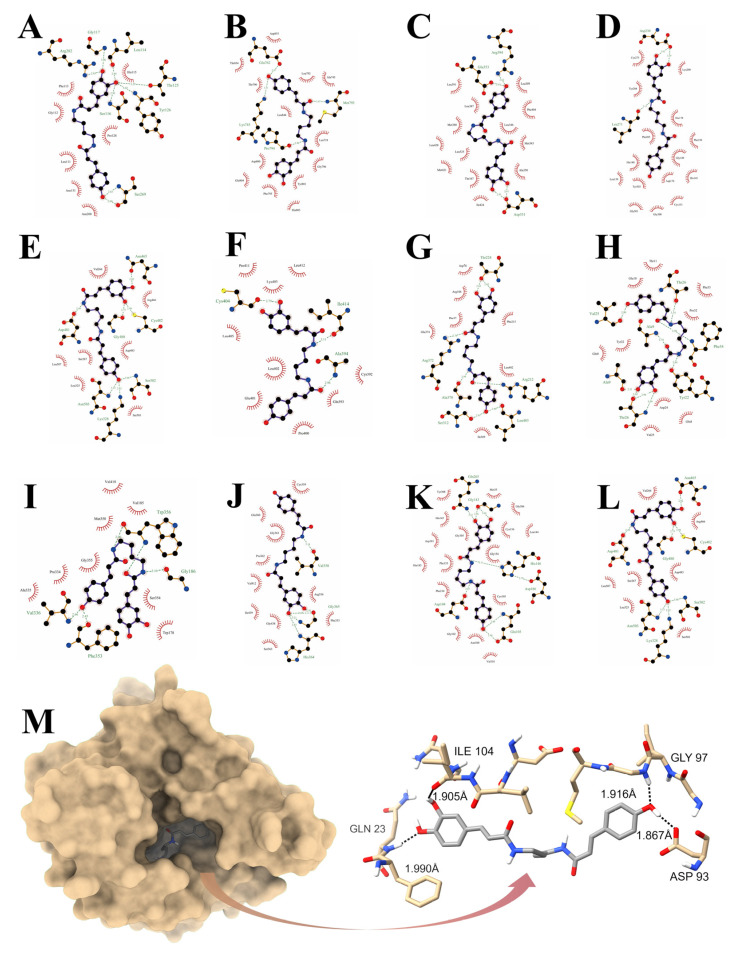
Reverse virtual docking: 2D interaction diagram of PCC and TP53 (**A**); 2D interaction diagram of PCC and EGFR (**B**); 2D interaction diagram of PCC and ESR1 (**C**); 2D interaction diagram of PCC and HDAC1 (**D**); 2D interaction diagram of PCC and SIRT1 (**E**); 2D interaction diagram of PCC and ICAM1 (**F**); 2D interaction diagram of PCC and CYP3A4 (**G**); 2D interaction diagram of PCC and APP (**H**); 2D interaction diagram of PCC and RELA (**I**); 2D interaction diagram of PCC and NFKB1 (**J**); 2D interaction diagram of PCC and HDAC2 (**K**); 2D interaction diagram of PCC and STAT1 (**L**); 2D and 3D interaction diagram of PCC and HSP90AA1 (**M**).

To clarify the binding affinity of PCC for 13 hub targets, a reverse virtual docking procedure was implemented via AutoDock. The parameters of the reverse virtual docking, as well as the ligand-receptor binding energies, are shown in Table 3. The interactions between the PCC and each potential target are shown in Figure 5. In this study, the greatest binding energy (−10.74 kcal/mol) was attributed to the complex formed by PCC with HSP90AA1, in which Gln23, Asp93, Gly97, and Ile104 of the N-terminal domain (NTD) perform critical actions (Figure 5M).

### 3.7. Molecular Dynamics Simulations

**Figure 6:**
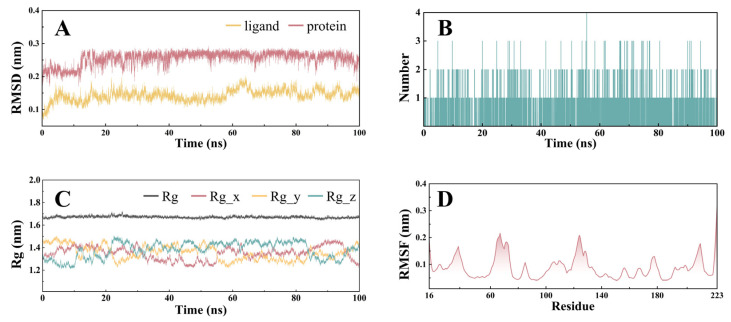
Molecular dynamics simulation: RMSD analysis of the PCC-HSP90AA1 complex (**A**); H-bond analysis of the PCC-HSP90AA1 complex (**B**); Rg analysis of the PCC-HSP90AA1 complex (**C**); and RMSF analysis of the PCC-HSP90AA1 complex (**D**).

The result was analyzed after the completion of the 100 ns molecular dynamics simulation. Figure 6A shows that the RMSD values of the ligand-receptor complex stabilized after 15 ns, with fluctuations consistently being within 0.1 nm (Figure 6A). The H-bond analysis revealed that PCC could provide at least one hydrogen bond to advance its binding to HSP90AA1 during the 100 ns simulation (Figure 6B). Additionally, the RG analysis implied that the fluctuation range of the complex remained under 0.1 nm over the 100 ns simulation (Figure 6C), whereas the RMSF analysis revealed some flexible residue fragments in the receptor that may interact with the ligand (Figure 6D). Taken together, these results support the fact that PCC can form a stable complex structure with HSP90AA1 and thus, perform its biological functions.

### 3.8. PCC Bound to HSP90AA1

**Figure 7:**
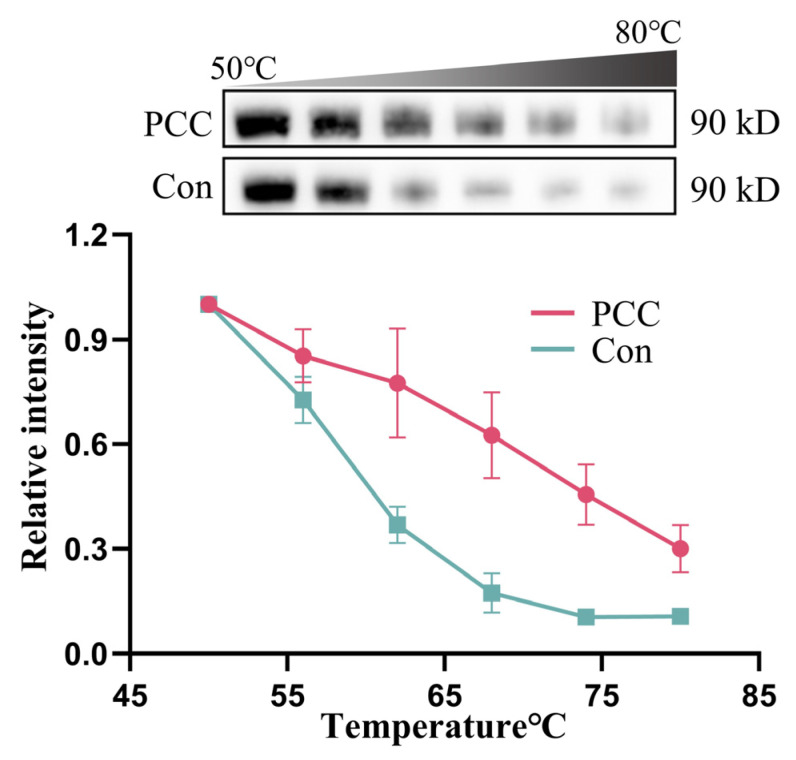
Identification of PCC binding to target protein HSP90AA1 via CETSA.

CETSA results indicated that the complex formed by PCC binding to HSP90AA1 reduced the temperature sensitivity of HSP90AA1 (Figure 7). Within the temperature range of 50–80 °C, although the relative band intensity of HSP90AA1 in PCC-treated cell lysates gradually decreased with increasing temperature, the stability of this protein significantly improved, compared with that of the control group at the same temperature.

### 3.9. In Vitro Evaluation

**Figure 8:**
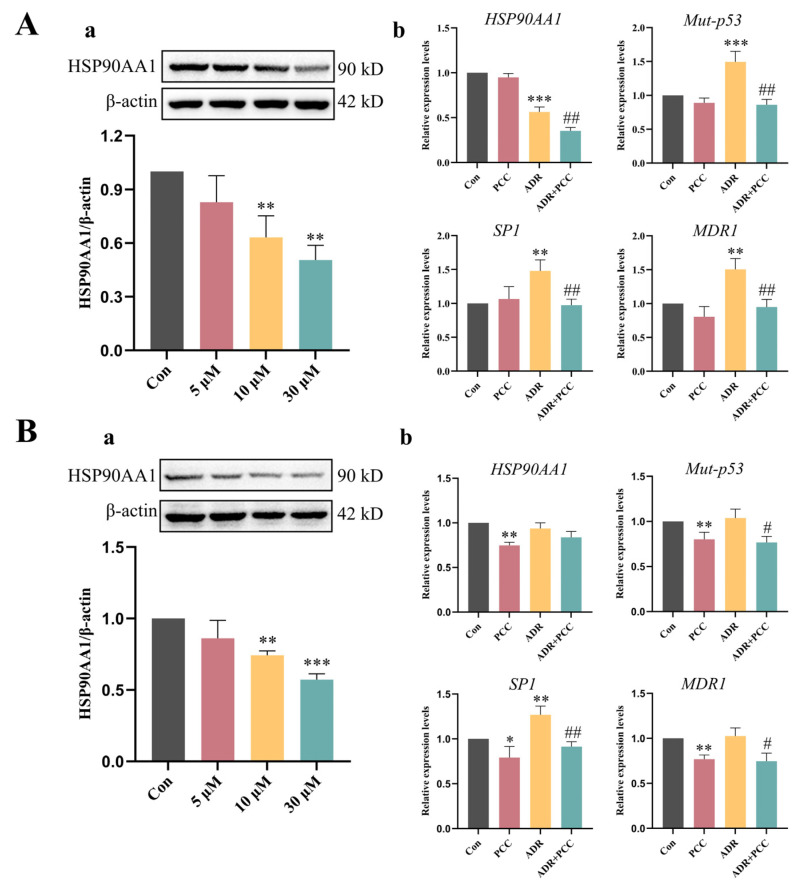
Semiquantitative analysis of HSP90AA1 via Western blot of HepG2 cells (**Aa**). Effect of PCC on the expression levels of *HSP90AA1*, *Mut-p53*, *SP1* and *MDR1* mRNAs in HepG2/ADR cells (**Ab**). Semiquantitative analysis of HSP90AA1 via Western blot of MCF-7 cells (**Ba**). Effect of PCC on the expression levels of *HSP90AA1*, *Mut-p53*, *SP1* and *MDR1* mRNAs in MCF-7/ADR cells (**Bb**). Compared to the Con group, * represents *p* < 0.05, ** represents *p* < 0.01, *** represents *p* < 0.001. Compared to the ADR group, # represents *p* < 0.05, ## represents *p* < 0.01.

After co-culturing with different concentrations of PCC for 24 h, the results showed that PCC dose-dependently decreased HSP90AA1 expression in both HepG2 and MCF-7 cells (Figure 8Aa,Ba). The cell viability was assessed via the CCK-8 assay (Figure S6). In HepG2 cells, PCC in the concentration range of 0–120 μM did not show significant cytotoxicity. In contrast, its treatment with 50 μM ADR resulted in a significant decrease in cell viability, with a reduction of 38.3%, compared with that of the control group. When 50 μM ADR was co-treated with 5 μM PCC, the cell viability was reduced by 50.5%, compared with that of the control group. In MCF-7 cells, the cell viability was reduced by 41.7% after treating with 50 μM ADR for 24 h. When 5 μM PCC was combined with 50 μM ADR, the cell viability decreased by 45.7%. These results suggested that PCC promoted apoptosis when in combination with ADR. Through the use of a fixed concentration of ADR (50 μM) with PCC (10 μM) to treat HepG2 cells and MCF-7 cells, it was found that PCC reduced the high transcription levels of the *SP1* and *MDR1* genes induced by ADR, while also inhibiting the transcription of *HSP90AA1* and *Mut-p53* (Figure 8Ab,Bb). In summary, in vitro experiments demonstrated that PCC acts as an HSP90AA1 inhibitor, promoting ADR efficacy and exerting anticancer activity by influencing the Mut-p53/SP1/MDR1 pathway. Notably, the effects of PCC are slightly more pronounced in HepG2 cells, compared to MCF-7 cells.

### 3.10. ADMET and Physicochemical Properties of PCC

The physicochemical properties of PCC were analyzed according to the Lipinski rule of five (Table 4). The appropriate molecular weight, number of hydrogen bond donors and acceptors, and LogP value all suggest that PCC has great potential as a pharmaceutical candidate. The results of the ADMET prediction are enumerated in Table 5. The PCC showed acceptable absorption, with a Caco-2 permeability value of −5.052. The cytochrome P450 (CYP) enzyme family, which catalyzes the oxidative metabolism of various endogenous and exogenous substances, was analyzed in terms of substrate and inhibitor predictions, providing insights for future targeted therapies involving PCC. Furthermore, the toxicity analysis of PCC revealed no significant toxicity, including DILI, rat oral acute toxicity, human hepatotoxicity, and immunotoxicity.

## 4. Discussion

Thousands of years of continuous evolution have led to the production of a diverse array of secondary metabolites in plants. These secondary metabolites have acquired various biological functions through their interactions with biological systems [29]. Consequently, in the face of considerable challenges in drug development, researchers often tend to look for countermeasures from the secondary metabolites of plants. *S. tangutica* is a Tibetan medicinal herb with a long medicinal history, which has been characterized by its excellent antioxidant, anti-inflammatory, anti-hepatic injury and antitumor properties in modern pharmacology [30,31]. In this study, a natural alkaloid, named *N*-*p*-coumaroyl-*N*’-caffeoylputrescine (PCC), was isolated from the Tibetan medicinal plant *S. tangutica* using a combined medium-pressure and high-performance chromatography approach. A literature search revealed that the structure and spectral information of PCC were first recorded in 2011 from the ethyl acetate fraction of *Exochorda racemosa* [28]. However, in the subsequent decade, no further reports have identified PCC from natural products, and its bioactivity and molecular targets remain entirely unexplored.

The biological activity and targets of small molecular compounds are often the primary challenges to be addressed in the drug development process. DARTS allows for the identification of binding targets of small molecules in biological systems without altering their structure or activity. In addition, as far as online databases are concerned, the PharmMapper database, which is based on pharmacophore matching; the STP and SuperNet databases, which are based on structure-activity relationships; and the Super-Pred database, which is based on machine learning, retrieve the potential targets of small molecule compounds from different dimensions, and the organic integration of these databases can mine the potential targets of small molecules as much as possible and characterize the complex interactions between them [32,33]. Our research confirmed that such a comprehensive strategy is applicable to PCC. The 317 common targets identified by the combination of multiple databases and DARTS were further screened (PPI network and reverse virtual docking), and HSP90AA1 was finally targeted as PCC’s target, which was also validated by subsequent experiments.

Heat shock protein 90 (HSP90) is a highly conserved ATP-dependent molecular chaperone in eukaryotes, which is important for maintaining cellular homeostasis and regulating the cell cycle. The expression levels of HSP90 under stress conditions are 2–6 times higher than those under normal conditions [34], which may be attributed to the overexpression of HSP90α (a subtype of HSP90). In contrast to HSP90β, HSP90α is encoded by the *HSP90AA1* gene and is often abnormally expressed in various diseases [35,36]. Oxidative stress is an essential trigger for the aberrant expression of HSP90AA1, and some antioxidants regulate the oxidative homeostasis of the body while driving HSP90AA1 back to the normal level or interfering with the function of client proteins by targeting HSP90AA1 [37]. Our work, firstly, clarified that PCC has an excellent antioxidant activity, and further target validation experiments found that the thermal stability of HSP90AA1 was improved after binding to PCC, implying that HSP90AA1 is the molecular target of PCC. The N-terminal domain (NTD) of HSP90AA1 contains an ATP-binding site (Bergerat fold) [38]. Molecular docking and dynamics simulation revealed that PCC interacts with residues Gln23, Asp93, Gly97, and Ile104 within the ATP-binding site, causing steric hindrance that disrupts ATPase activity and prevents transient dimerization. This misfolded conformation ultimately leads to the degradation and functional loss of its client proteins. Bioinformatics analyses and data mining suggest that PCC has promising applications in cancer by affecting the activity and expression of HSP90AA1, subsequently interacting with TP53, EGFR, and ESR1 (client proteins of HSP90AA1), triggering a cascade reaction.

For cancer, pharmacologic interventions remain the primary approach for its treatment; however, the resistance generated in this process is rapidly attracting the attention of scientists [39]. Moreover, the resistance generated by one anticancer drug is extremely likely to reduce the sensitivity of other drugs, and this manifestation of multidrug resistance (MDR) is one of the major reasons for the failure of cancer treatment [40,41]. Multi-drug resistance gene-1 (MDR1) is the crux in addressing drug resistance in many malignant tumors, and its encoded P-pg (P-glycoprotein) is able to pump intracellular drugs out of the cytoplasm by hydrolyzing ATP for its own energy supply [42]. In addition, previous studies have also reported excessive HSP90AA1 stature in studies of MDR [43], whose client protein mutant p53 (Mut-p53) in malignant tumors reduces drug efficacy (off-target), and it can also activate the transcription factor SP1 (specificity protein 1), which promotes the transcription and expression of MDR1 [44,45]. In this study, PCC was able to reduce the expression of HSP90AA1 and had no significant impact on the viability of HepG2 and MCF-7 cells, suggesting that the anticancer activity of PCC may not be directly related to apoptosis. However, PCC, in combination with ADR, reduced the viability of tumor cells more than ADR alone did; reversed the high transcript levels of *Mut-p53*, *SP1* and *MDR1* caused by ADR and increased the cell sensitivity to drugs. As a prominent tumor suppressor gene, TP53 is also one of the hub targets of PCC, and its encoded protein P53 is often present in its mutant form in malignant tumors [46]. PCC was shown to reduce the transcriptional levels of Mut-p53, which implies that the synergistic effect with other targets while PCC exerts its biological function through HSP90AA1 is also an important factor to consider. Overall, these findings imply that PCC, as a small-molecule inhibitor of the chaperone protein HSP90AA1, is able to further expand the potency of ADR for antitumor purposes through the Mut-p53/SP1/MDR1 pathway, demonstrating a significant therapeutic potential.

In conclusion, this study integrated network pharmacology and DARTS into a reproducible strategy to clarify HSP90AA1 as the target of the natural alkaloid PCC, with Gln23, Asp93, Gly97, and Ile104 being key residues. In future work, crystallography and site-directed mutagenesis will be emphasized in the exploration of the interaction mechanism and structure-activity relationship between HSP90AA1 and PCC. Additionally, taking the biological function of HSP90AA1 as an entry point, the potential of PCC in cancer therapy was explored in this work. Given that PCC is an effective HSP90AA1 inhibitor, a more comprehensive in vitro and in vivo experimental design will be worth considering to further explore its medicinal potential and evaluate its pharmacokinetics.

## 5. Conclusions

This study utilized DARTS technology, combined with online databases, to fish for the protein targets of PCC, a natural alkaloid from *S. tangutica*. A total of 317 common targets were identified, which were further analyzed using bioinformatics and reverse virtual screening. Ultimately, HSP90AA1 was confirmed as the target of PCC, with a binding energy as low as −10.74 kcal/mol. Our findings suggested that PCC acts as a potent antioxidant, along with being a promising candidate for cancer treatment. Follow-up in vitro experiments showed that the combination of PCC with ADR significantly reduces the expression of genes associated with drug resistance. In conclusion, this study offers valuable insights into the development of PCC as a lead compound; meanwhile, such a strategy also provides robust assistance for future bioactivity and protein target studies of natural products.

## Figures and Tables

**Table 1:** Sequences of primers used for qPCR.

Gene	Primer Sequence
*β-actin*	5′-AAGAGATGGCCACGGCTGCT-3’ 5′-GACTCGTCATACTCCTGCTTG-3’
*HSP90AA1*	5′-GTCTGGGTATCGGAAAGCAAG-3’ 5′-CTGAGGGGTGGGGATGATGTC-3’
*Mut-p53*	5′-CCTCAGCATCTTATCCGAGTGG-3’ 5′-TGGATGGTGGTACAGTCAGAGC-3’
*MDR1*	5′-CCCATCATTGCAATAGCAGG-3’ 5′-GTTCAAACTTCTGCTCCTGA-3’
*SP1*	5′-GCTATGCCAAACCTACTCC-3’ 5′-CTGTAGCCCACTGACCT-3’

**Table 2:** ^1^H-NMR and ^13^C-NMR data of PCC.

Atom Number	^1^H-NMR	^13^C-NMR
1	7.32	138.5
2	6.3	118.6
3	-	165.3
4	7.97	-
5	3.17	38.3
6	1.46	26.8
7	1.46	26.8
8	3.17	38.3
9	7.97	-
10	-	165.3
11	6.4	118.8
12	7.23	138.9
1′	-	126.4
2′	6.93	113.8
3′	-	145.5
4′	-	147.2
5′	6.74	113.8
6′	6.83	120.3
1″	-	126
2″	7.38	129.1
3″	6.79	115.7
4″	-	158.8
5″	6.79	115.7
6″	7.38	129.1

**Table 3:** Reverse virtual docking parameters and results.

Protein	Number of Points	Center Grid Box	Spacing	Binding Energy
TP53 ID: 2XWR	X_dimension = 88 Y_dimension = 108 Z_dimension = 76	X_center = −42.03 Y_center = 10.36 Z_center = −53.92	0.408	−9.41 kcal/mol
EGFR ID: 4G5J	X_dimension = 90 Y_dimension = 92 Z_dimension = 72	X_center = 63.19 Y_center = 8.73 Z_center = −22.84	0.614	−6.94 kcal/mol
ESR1 ID: 7UJW	X_dimension = 118 Y_dimension = 102 Z_dimension = 120	X_center = −4.09 Y_center = 19.58 Z_center = 71.2	0.375	−8.96 kcal/mol
HSP90AA1 ID: 3O0I	X_dimension = 82 Y_dimension = 70 Z_dimension = 92	X_center = −0.48 Y_center = −11.34 Z_center = −30.30	0.51	−10.74 kcal/mol
HDAC1 ID: 4BKX	X_dimension = 90 Y_dimension = 90 Z_dimension = 74	X_center = −61.35 Y_center = −16.19 Z_center = −5.84	0.68	−7.14 kcal/mol
SIRT1 ID: 5BTR	X_dimension = 92 Y_dimension = 82 Z_dimension = 106	X_center = −16.77 Y_center = 66.21 Z_center = 4.02	0.653	−8.21 kcal/mol
NFKB1 ID: 3GUT	X_dimension =102 Y_dimension = 64 Z_dimension = 100	X_center = 45.02 Y_center = −22.83 Z_center = 47.6	0.808	−5.67 kcal/mol
CYP3A4 ID: 1TQN	X_dimension = 58 Y_dimension = 106 Z_dimension = 94	X_center = −18.93 Y_center = −25.25 Z_center = −13.91	0.61	−9.61 kcal/mol
APP ID: 1AAP	X_dimension = 80 Y_dimension = 84 Z_dimension = 98	X_center = 16.73 Y_center = 19.36 Z_center = 36.51	0.375	−5.92 kcal/mol
RELA ID: 6POZ	X_dimension = 80 Y_dimension = 76 Z_dimension = 98	X_center = 102.83 Y_center = −14.07 Z_center = −21.91	0.669	−9.12 kcal/mol
ICAM1 ID: 1P53	X_dimension = 119 Y_dimension = 94 Z_dimension = 54	X_center = 9.35 Y_center = 150.7 Z_center = 46.94	0.831	−5.54 kcal/mol
HDAC2 ID: 3MAX	X_dimension = 78 Y_dimension = 92 Z_dimension = 88	X_center = 90.83 Y_center = 40.31 Z_center = −41.94	0.525	−9.11 kcal/mol
STAT1 ID: 1YVL	X_dimension = 104 Y_dimension = 60 Z_dimension = 108	X_center = −12.28 Y_center = −44.74 Z_center = 84.37	0.986	−4.85 kcal/mol

**Table 4:** Physicochemical properties of PCC.

Property	Value	Optimal Value
MW	396.17	100~600
nHD	5	0~7
nHA	7	0~12
nRot	11	0~11
nRing	2	0~6
MaxRing	6	0~18
nHet	7	1~15
TPSA	118.89	0~140
LogS	−3.064	-
LogP	1.643	-
LogD	1.954	-
Synth	2	<6

**Table 5:** ADMET prediction of PCC.

Property	Value	Optimal Value
Caco-2 Permeability	−5.052	>−5.15
HIA	0.961	-
BBB	0	-
VDss	−0.715	0.04~20
HLM stability	0.002	0
CYP1A2 inhibitor	1	0
CYP1A2 substrate	0	0
CYP2D6 inhibitor	0.018	0
CYP2D6 substrate	0.281	0
CYP3A4 inhibitor	0	0
CYP3A4 substrate	0	0
CLplasma	12.563	>15
DILI	0.612	<1
Rat Oral Acute Toxicity	0.025	<1
Carcinogenicity	0.055	<1
Human Hepatotoxicity	0.588	<1
Drug-induced Nephrotoxicity	0.041	<1
Drug-induced Neurotoxicity	0.596	<1
Hematotoxicity	0.005	<1
Immunotoxicity	0.023	<1

## Data Availability

Data are contained within the article and Supplementary Materials.

## Supplementary Materials

The following supporting information can be downloaded at: https://www.mdpi.com/article/10.3390/antiox14010012/s1, Figure S1: ESI-MS spectrum data of Fr4-5-1; Figure S2: ^1^H-NMR data of Fr4-5-1 (DMSO-d6); Figure S3: ^13^C-NMR data of Fr4-5-1 (DMSO-d6); Figure S4: HSQC data of Fr4-5-1; Figure S5: HMBC data of Fr4-5-1; Figure S6: Cell viability assay of HepG2 (A) and MCF-7cells (B); Table S1: Potential target of PCC; Original data S1: Darts; Original data S2: molecular dynamics simulation.
