# Supplementary material for: Drug Target Investigation of N-p-Coumaroyl-N’-Caffeoylputrescine, a Naturally-Occurring Alkaloid Derived from Saxifraga tangutica
Source: Antioxidants (Basel). 2024 Dec 25;14(1):12. doi: 10.3390/antiox14010012 (PMC11762362; doi:10.3390/antiox14010012)
Supplement: Supplementary file 1 [file antioxidants-14-00012-s001.zip › antioxidants-3331878-supplementary.pdf]

Supplementary Material

Drug targets investigation of *N–p–*coumaroyl–*N′*–caffeoylputrescine, a naturally alkaloid derived from *Saxifraga tangutica*

Chuang Liu[1], Jun Dang[2, *], Minchen Wu[1, *]

[1]*School of Biotechnology, Jiangnan University, Wuxi 214122, China*

[2]*Key Laboratory of Tibetan Medicine Research, Northwest Institute of Plateau Biology, Chinese Academy of Sciences, Xining, 810000, China*

[*]Corresponding authors.
E–mail address: dangjun@nwipb.cas.cn (Jun Dang), biowmc@126.com (Minchen Wu)

Table of Contents

Figure S1 ESI−MS spectrum data of Fr4−5−1 3

Figure S2 ^{1}H−NMR data of Fr4−5−1 (DMSO−d_6).......... 3

Figure S3 ^{13}C−NMR data of Fr4−5−1 (DMSO−d_6).......... 4

Figure S4 HSQC data of Fr4−5−1 4

Figure S5 HMBC data of Fr4−5−1 5

Figure S6 Cell viability assay of HepG2 (A) and MCF-7cells (B) 5

Table S1. Potential target of PCC.......... 6

Original data 1. Darts 6

Original data 2. molecular dynamics simulation 6

Figure S1 ESI−MS spectrum data of Fr4−5−1

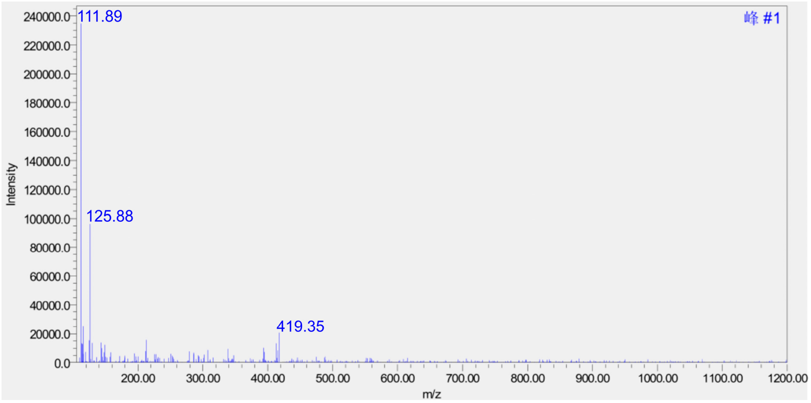

Figure S2 ^{1}H−NMR data of Fr4−5−1 (DMSO−d_6)

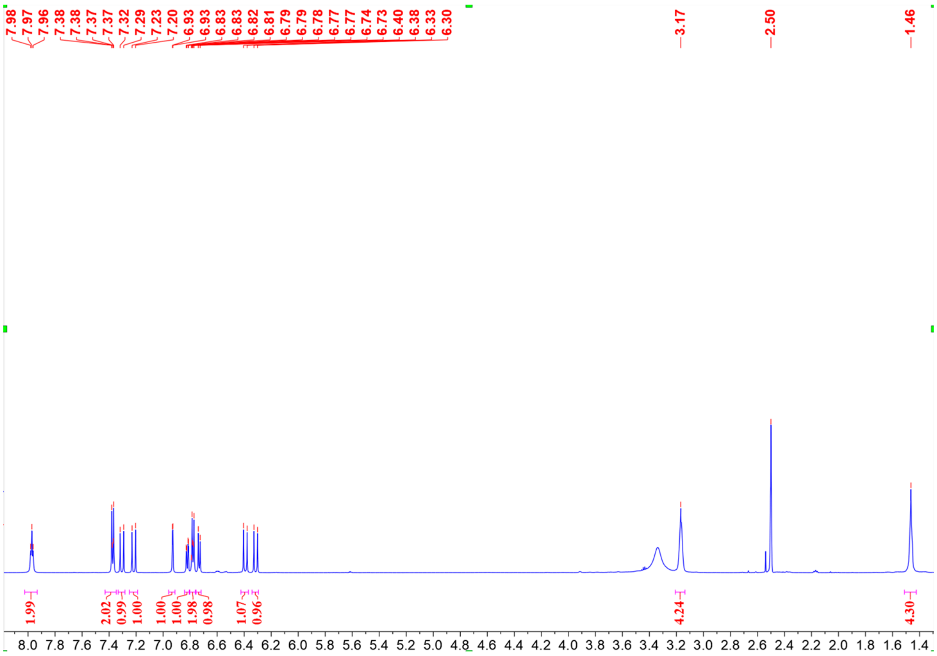

Figure S3 ^{13}C−NMR data of Fr4−5−1 (DMSO−d_6)

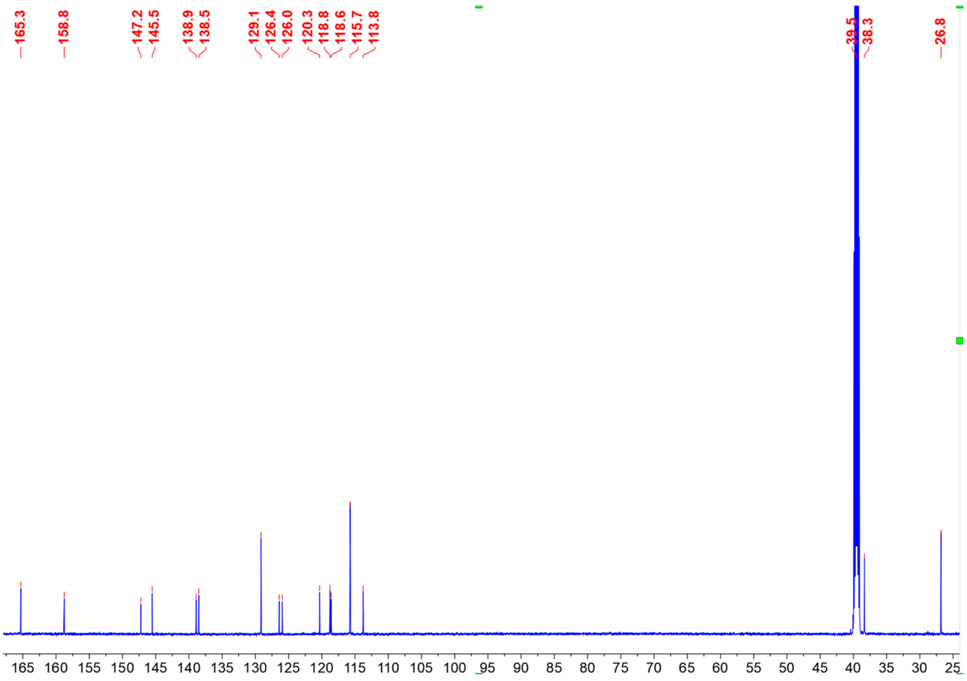

Figure S4 HSQC data of Fr4−5−1

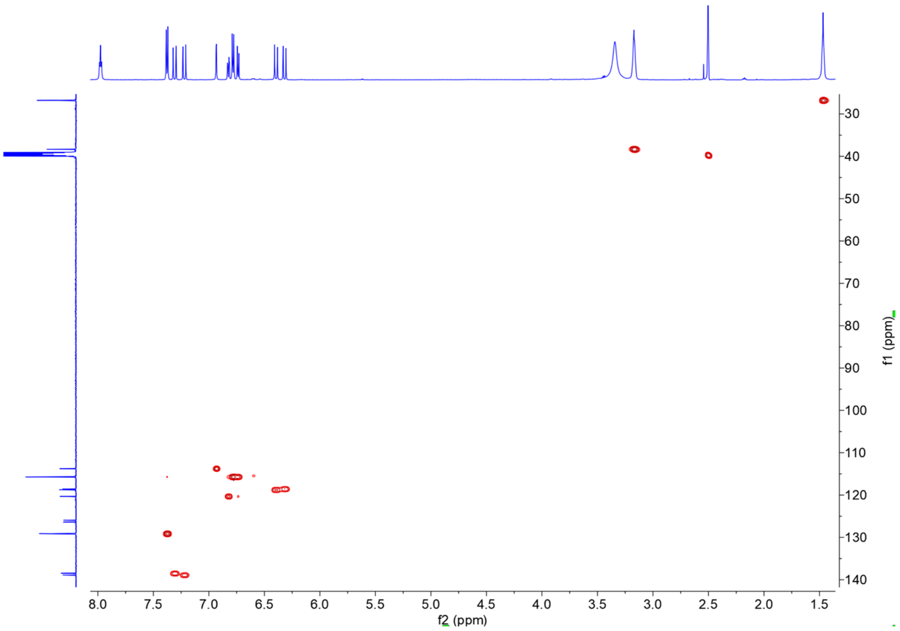

Figure S5 HMBC data of Fr4–5–1

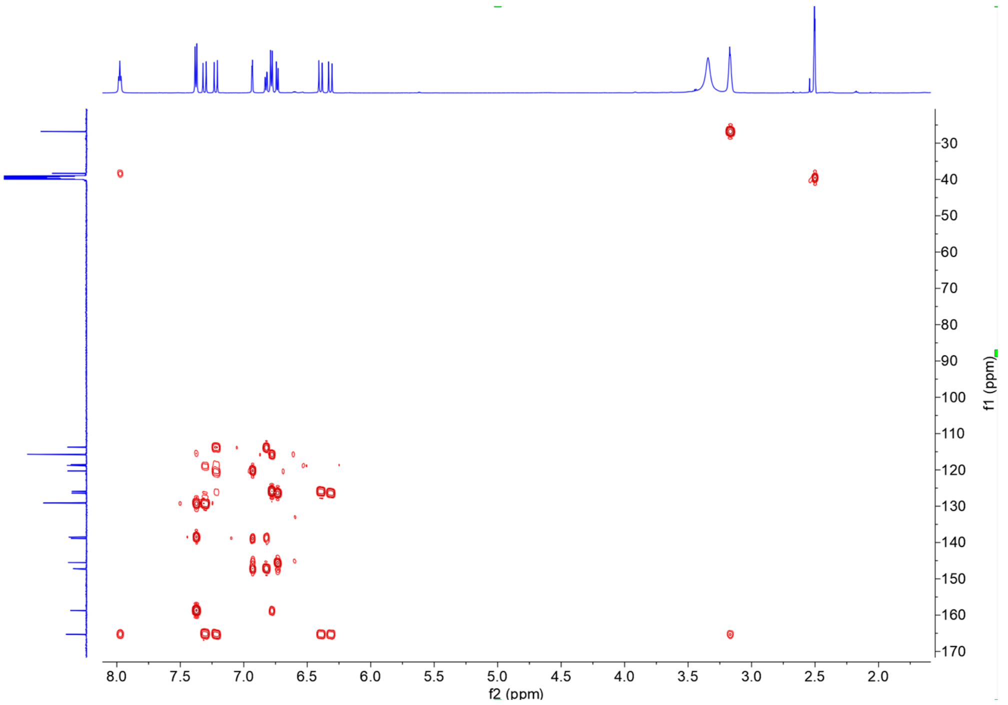

Figure S6 Cell viability assay of HepG2 (A) and MCF-7cells (B)

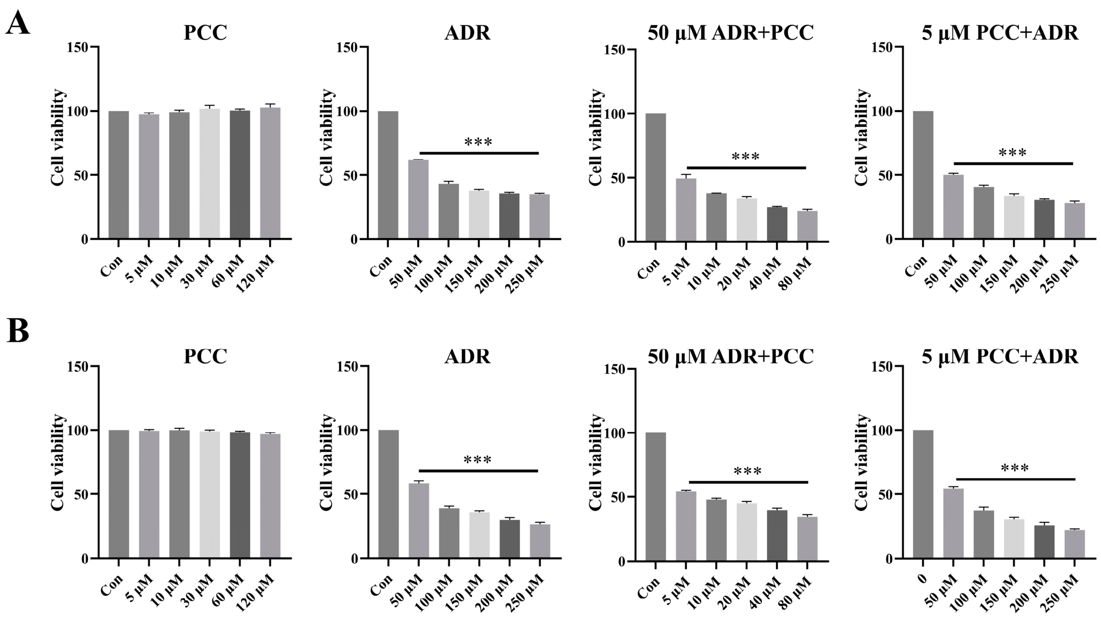

Table S1. Potential target of PCC

Source	Targets								
DARTS	Heatr5a	Spcs3	Kiaa1522	Mvb12a	Ppp1r13l	Slx9	Bcl10	Clpx	Etl4
	Naa16	Cbl	Map4k4	Nucks1	Tmsb10	Mettl2	Zcchc8	Serpinb6	Tmem106a
	Nup205	Nolc1	Tubb2a	Pdap1	Mrpl43	Zc3hc1	Srsf2	Bid	Cyc1
	Mark2	Hirip3	Fau	Mapre2	Ybx1	Spats2	Esam	Wipi2	Trim14
	Pdzd11	Jak1	Dhx32	Nsfl1c	Vdac1	Ufm1	Tbl2	Zw10	Polr2k
	Agap1	Wdr18	Pten	Tspan4	Ppcs	Snx7	Eri1	Ano10	Scd1
	Gyg1	Plin2	Trappc4	Gmpr2	Dhrs7	Pacsin3	Slc39a7	Xrn1	Tsen34
	Ceacam1								
PharMapper	CBL	VAV2	RND1	VWF	DHPS	HBP1	IFNGR1	GALK1	CNDP1
	TNPO1	TP53	THOC1	PHGDH	EXOSC9	HAT1	HK2	NAE1	AMD1
	VPS26A	CD1A	IDE	UTRN	FHIT	DGKA	HMBOX1	OPG111	OXSR1
	NCK2	HASPIN	GALNT10	RIMBP2	TDP1	CPSF3	SEC24C	R3HDM2	C3
	UL30	PA2G4	TRNT1	GNPNAT1	AKR1C3	CKM	BCAT2	ACY1	PCTP
	HSD17B14	CYP2C8	SORD	GSR					
Super-PRED	APEX1	HSD17B10	TDP1	CTSD	GUSB	SLC6A5	TTR	NFE2L2	HDAC7
	HDAC5	KLF5	PSMB1	NFKB1	TRIM24	GLRA1	ALOX12	PLK4	CACNA1H
	NR1I2	TAOK1	TOP2A	QRFPR	STAT1	CHRNB4	FPR2	TAOK3	ESR1
	GBA1	HTR2C	CLK4	SLC2A1	XDH	NTSR2	ESR2	CYP2A6	ACVR1B
	GSTP1	NQO2	PSMB2	MAP2K2	RORB	BMP2K	GRK5	FCGRT	RPS6KA5
	PIN1	THRA	PIK3R1	DPP8	LGALS3	FPRL2	CYP3A4	HDAC9	ADORA2B
	GRIN1	DPP9	EPHB2	CDK5	CDC25B	KDM6B	F13A1	SCN3A	HDAC8
	SIRT3	IL23R	AOC3	HDAC2	PYGL	CXCR4	ADORA3	PDGFRB	NR3C2
	ADAM10	YES1	PDGFRA	AAK1	SLC1A2	P2RX4	MAOA	METAP2	KCNK9
	SLC1A1	CHRNA1	ADORA1	FPR1	CHEK1	RPS6KA3	TFPI	SLC1A3	KDM1A
	ABCC1	CSNK2B	NAMPT	NR4A1	ITGB1	S1PR3	MDM4	CHUK	MMP9
	CAPN1	SLC40A1	DPP7	S1PR4	MIF	CHRM3	PTK2B	AVPR1B	CCR2
	P2RY6	ULK3	CCR1	MARK4	SAE1	NOS2			
STP	EGFR	DNM1							
TargetNet	HDAC8	ESR1	AKR1B1	CA5A	ESR2	APP	MAOB	CA12	KCNA3
	CA14	MAOA	CA9	MIF	FOLH1	ALOX15	PLIN5	HDAC3	CXCR4
	HNF4A	HDAC4	MMP9	ABCG2	CA7	GPR35	HDAC1	HDAC2	ADRA2C
	HDAC6	CDC25B	CA5B	CA4	CA6	ALPL	TLR9	PTPN1	PTGFR
	MMP2	RIPK2	ALOX5	ICAM1	PTGS1	CTDSP1	RELA	MMP1	CA2
	HSD17B3	DRD3	PLIN1	CACNA1B	OPRD1	AHR	PTPN22	CYP1A2	SERPINE1
	DUSP3	PRKCE	EGFR	PRKCG	GRIN2B	HSD17B1	CA13	TAAR1	STS
	MDM2	PYGL	BCL2A1	DNMT1	TERT	S1PR2	PTGER2	ACHE	ATP4A
	SELE	MGLL	LTB4R	APOBEC3A	PIM1	METAP2	DRD2	TRPV1	RPS6KA3
	CYP19A1	SIRT1	CES2	CA1	MMP12	PTPN7	OPRM1	NR2F2	HTR1E
	HSP90AA1	PTPN2	SIRT2	RARG	APOBEC3G	THRA	PLA2G1B	HSD17B2	S1PR4
	OPRK1	CCR2	DRD1	ITGAL	CCKAR	HCAR2	CYP2C19		

Original data 1. Darts

darts probe.xlsx

Original data 2. molecular dynamics simulation

hbnum.xvg rmsf.xvg rmsd_ligand.xvg rmsd_protein.xvg gyrate.xvg
